# Documenting the Density of Subtidal Marine Debris across Multiple Marine and Coastal Habitats

**DOI:** 10.1371/journal.pone.0094593

**Published:** 2014-04-17

**Authors:** Stephen D. A. Smith, Robert J. Edgar

**Affiliations:** National Marine Science Centre, Southern Cross University, Coffs Harbour, New South Wales, Australia; The Evergreen State College, United States of America

## Abstract

Marine debris is recognised globally as a key threatening process to marine life, but efforts to address the issue are hampered by the lack of data for many marine habitats. By developing standardised protocols and providing training in their application, we worked with >300 volunteer divers from 11 underwater research groups to document the scale of the subtidal marine debris problem at 120 sites across >1000 km of the coast of NSW, Australia. Sampling consisted of replicated 25×5 m transects in which all debris was identified, counted, and, where appropriate, removed. Sites ranged from estuarine settings adjacent to major population centres, to offshore islands within marine parks. Estuaries and embayments were consistently found to be the most contaminated habitats. Fishing-related items (and especially monofilament and braided fishing line) were most prevalent at the majority of sites, although food and drink items were important contributors at sites adjacent to population centres. The results identified damaging interactions between marine debris and marine biota at some key locations, highlighting the need for management intervention to ensure habitat sustainability. This study reinforces the important contribution that volunteers can make to assessing conservation issues requiring broad-scale data collection. In this case, citizen scientists delivered data that will inform, and help to prioritise, management approaches at both statewide and local scales. These initial data also provide an important baseline for longer-term, volunteer-based monitoring programs.

## Introduction

Marine debris is a growing problem with impacts on marine life [1], [2], aesthetics [3]–[5], global economies [6] and ecosystem sustainability [7]. Impacts on wildlife have received considerable attention in the recent past with an every-increasing list of species documented to sustain negative effects from interactions with debris [8], [9]. The primary impacts include ingestion and entanglement, both of which may lead to mortality [2]. Primarily because many susceptible taxa are charismatic, or listed as threatened (e.g. turtles [10]–[12], pinnipeds [13]–[15], cetaceans [16], [17] and birds [18]–[20]), marine debris is regarded as a key threatening process to marine fauna by conservation organisations and government agencies worldwide (e.g. [21].

Debris is prevalent in all marine environments [22], although quantification has focused primarily on accessible habitats such as beaches, estuaries and the ocean surface. In subtidal benthic habitats, there is clear evidence that the presence of debris can result in the mortality of vulnerable taxa, such as hard and soft corals [23], [24], with flow-on effects to broader biotic diversity [25], [26]. The fact that diverse habitats are often targeted by activities that generate debris, such as recreational fishing, highlights the complex challenges associated with their effective conservation management [27].

While the huge scale of the marine debris issue has been recognised, a consistent approach to dealing with it has yet to be developed and implemented. In the meantime, and especially in developed countries, community education, which often includes targeted and regular clean-up activities [28]–[31], is widely used to take local steps to combat this global issue. In many cases, broad-scale monitoring can only be carried out where much of the workforce comprises volunteers [31]–[33], and guidelines have been developed with respect to their training and management [34]. Mainly because of access, most community-based clean-up events focus on intertidal areas in marine and estuarine habitats, and only recently have activities extended more broadly into subtidal habitats through international initiatives such as Project AWARE, and locally organised events, mostly at popular dive locations [24], [35], [36].

In New South Wales (NSW), Australia, an increasing number of non-government organisations are promoting debris awareness and clean-up activities (e.g. Clean Up Australia Day, Two Hands Project, Take 3, Tangaoroa Blue) focusing primarily on intertidal habitats. Divers also regularly participate in underwater clean-ups as part of organised activities through dive clubs and dive shops, often timed to coincide with national (e.g. Clean Up Australia Day) or international (International Clean Up Day) events. However, while broad records are generally kept of the type and total quantities of debris removed, seldom are data collected in a sufficiently rigorous way to enable quantitative comparisons between sites, or over time at the same sites. In response to this, the Underwater Volunteers NSW program was established to promote the use of standardised protocols by volunteers, with the objectives of providing accurate and comparable data for use by managing authorities. One of the first outcomes of this initiative was the introduction of a standardised method for surveys of subtidal marine debris, with subsequent application by 11 underwater volunteer groups across the state.

In this initial analysis of the data set, we provide a broad overview of debris loads at 120 sites across the NSW coastline. In particular, we focus on the prevalence of different types of debris and the contribution of different activities to total debris loads. We also the use the data to make broad observations about relative debris loads in discrete marine habitat types and to identify pressing management issues at both large (statewide) and small (local) scales.

## Methods

Work in marine protected areas was carried out under permits issued by the NSW Department of Primary Industries (Fisheries); however, no specific permissions were required for most locations. The field studies did not involve endangered or protected species.

Marine debris surveys have been conducted at a range of subtidal reefs in northern NSW since 2005 as part of a long-term program to monitor reef health [27]. We developed a simple process of gathering data by quantifying debris items in 4 replicate 25×5 m transects at each survey site [27]. By using the same methods with underwater volunteers, we planned to extend assessments of marine debris across a much broader area than would otherwise be possible – in this case, the entire coast of NSW (>1000 km). As volunteers had a range of experience and ability to work underwater, we developed a standard training procedure and all participants were required to successfully complete at least one training session prior to collecting data. Thus, all volunteers undertook classroom training to ensure they understood the context of the work and the need for standardised methods. Divers then worked with the training team, and volunteers who had already completed the training program, to: complete supervised underwater surveys using the standardised protocol; examine and classify the debris recorded; and record data in all requisite fields on the standardised data sheets (these materials are available on the UVNSW website – uvnsw.net.au).

Based on local knowledge, groups selected the most appropriate sites for conducting their surveys. Wherever possible, 4 transects were deployed at each site (this was not always possible due to tidal currents or other diving-related factors) and all debris occurring within these transects was recorded onto pre-printed data sheets. If practicable, debris items were removed: however, if items were habitat-forming, and their removal was likely to result in harm to marine biota, they were recorded but left *in situ*.

At least one member from each group either uploaded data directly onto the online UVNSW database (uvnsw.net.au), or submitted spreadsheets to the project team for data entry. Quality Assurance/Quality Control procedures are built into the data entry protocols ensuring high confidence in the resulting data set. Thus, data were read from the datasheet by one volunteer, entered onto the database by a second volunteer, and subsequently proofed by both.

**Figure 1:**
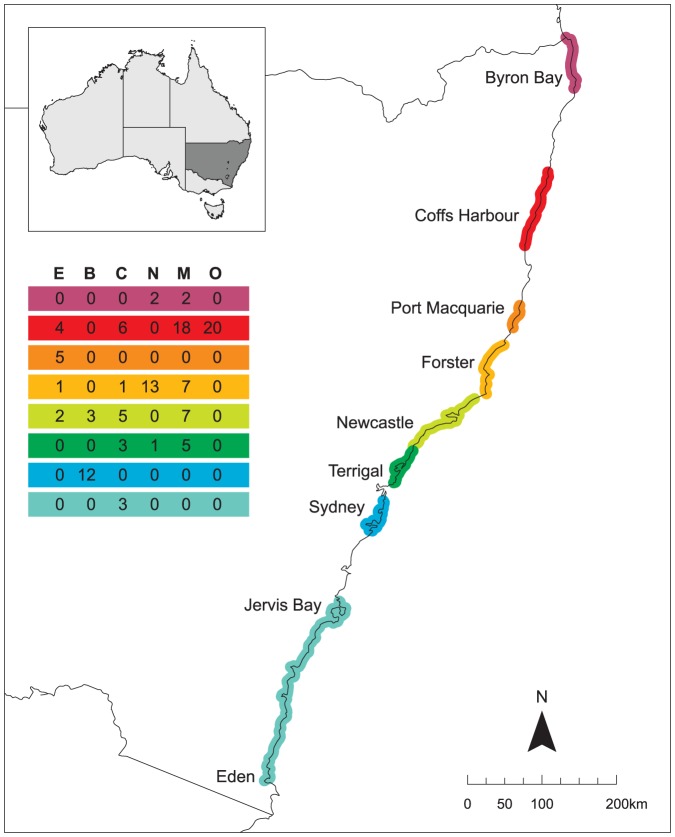
Map of the NSW coastline showing the regional coverage of the surveys. The table indicates the number of sites in each broad habitat category for each region: E =  estuary; B =  bay; C = coastal; N =  nearshore reef; M =  mid-shelf reef; O =  offshore reef.

To promote participation, we encouraged volunteer groups to collect data to address questions about marine debris at locally relevant scales. For this reason, groups had various objectives for their specific programs. The groups also had different capacities in terms of membership as well as access to different habitats and so it was not practicable to enforce an overarching design at the statewide scale. Thus, it was often the case that sampling within a specific region focused primarily on a limited number of habitat types (Fig. 1 and see below), or that some sites were targeted multiple times. For this analysis of standing stock of debris, we therefore used the first sample from sites where repeated sampling had been carried out. Data were collected from 120 sites from Cook Island in the north of the state, to Merimbula in the south (Fig. 1). The majority of the surveys were completed by groups in the central and mid-northern sections of the coast with relatively few sites sampled on the far north and far south coast. A total of 690 transects were deployed overall: this was reduced to 470 once temporal replicates were removed. For this overview of patterns and debris loads, we classified sites into 6 broad types based on their topographic setting and tidal regime: estuary (estuaries without a major embayment) (12 sites); bay (large embayments which experience tidal flow and freshwater influences – Sydney Harbour, Port Stephens – Fig. 1) (15 sites); coastal (sites within 150 m of the coast) (18 sites); nearshore reef (150 m–1.5 km from shore) (16 sites); mid-shelf reef (1.5–6 km from shore) (39 sites); and offshore reef (>6 km from shore) (20 sites). The classification for reefs is based on the categories currently used in the habitat classification system for subtidal habitats in the system of marine parks in NSW [37].

As the data were collected with different local objectives by each of the 11 groups, a comparison among regions was confounded by the lack of data from all habitat categories across regions (Fig. 1). For this reason, data analyses were necessarily broad and focused on patterns of difference across habitat types. We consequently used a 2-way nested design (habitat, site nested within habitat) and analysed the data using a range of multivariate statistical methods. To visually examine patterns in debris distribution, we averaged data across replicates within a site and generated a non-metric multidimensional scaling (nMDS) ordination based on a Bray-Curtis similarity matrix of raw data. Because there were a number of zero values, we added a dummy variable prior to generating the Bray-Curtis similarity matrix. The significance of differences in debris patterns were assessed using 2-way nested PERMANOVA of the full data set, with *post hoc* tests for significant main effects. The types of debris driving differences were further explored using similarity percentages (SIMPER) analysis and by superimposing vectors representing discriminant categories onto the nMDS plot.

The abundance (N_d_) and diversity (S_d_) of debris were analysed separately using univariate 2-way nested PERMANOVA of a matrix of Euclidean distances. Significant main effects were further explored using *post hoc* tests. All statistical analyses were performed using PRIMER 6+ PERMANOVA [38], [39].

## Results

Our surveys of 120 sites recorded a total of 2,986 items of marine debris. Debris loads (items per transect) ranged from 0 (210 of the 470 transects contained no debris) to 218 (the Pipeline, Port Stephens – Fig. 1). Plastic items were the most abundant (33% of the total), and mostly comprised of fishing monofilament (82% of plastic items and 27% of the total debris) which primarily originated from recreational fishing activities. Plastic fragments comprised the majority of the remainder of plastic items (10% of total debris) with plastic bags contributing a further 4% to total loads. Glass items contributed 20% of the total items and mainly comprised entire bottles (13% of total debris) and broken fragments (6% of total debris). A range of metal objects (18%), and items with mixed construction (18%), made up the majority of other items. Fishing was the primary source of most debris items (38% of the total), with food and drink accounting for a further 27% of the total.

**Figure 2:**
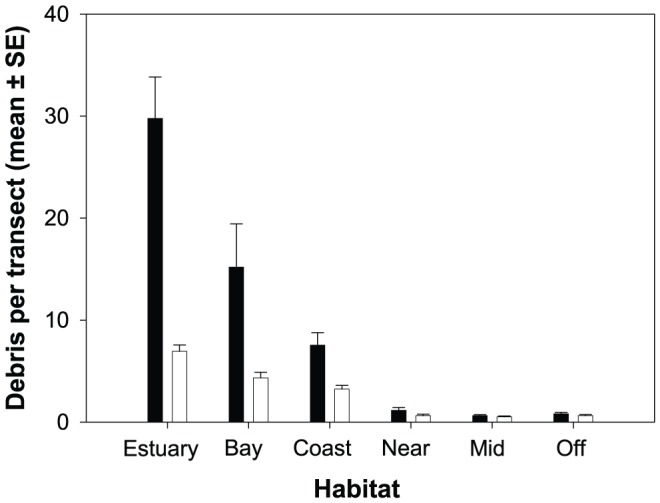
Mean (±SE) debris loads (filled bars) and diversity (unfilled bars) of debris items per transect averaged over all sites within each habitat category.

There was a clear difference in both the abundance and diversity of debris items by broad habitat type (Fig. 2). Thus, sites within estuaries and bays consistently returned the highest values for both metrics with loads reducing substantially >150 m from shore (the nearshore, mid-shelf and offshore reef habitats). This trend was found to be highly significant in the 2-way nested PERMANOVA (N_d_, P<0.001; S_d_, P<0.001) which also revealed a highly significant difference amongst sites nested within habitat type for both metrics (N_d_, P<0.001; S_d_, P<0.001). Pairwise contrasts (Table 1) revealed identical patterns for both debris abundance and diversity: values were significantly higher in estuaries than in all other habitats except bays; bays and coastal sites did not differ significantly but were significantly higher than the 3 reef categories; there were no differences among the reef categories.

**Table 1:** Summary of pairwise contrasts (PERMANOVA) amongst habitats for the multivariate tests of differences in debris structure, and for the univariate tests for abundance (N_d_) and diversity (S_d_). P(perm) values are shown with significant terms in bold font. E =  estuary; B =  bay; C = coastal; N =  nearshore reef; M =  mid-shelf reef; O =  offshore reef.

Comparison	Debris structure	N_d_	S_d_
E vs B	0.001	0.094	0.061
E vs C	<0.001	<0.001	0.001
E vs N	<0.001	<0.001	<0.001
E vs M	<0.001	<0.001	<0.001
E vs O	<0.001	<0.001	<0.001
B vs C	0.100	0.153	0.210
B vs N	<0.001	<0.001	<0.001
B vs M	<0.001	<0.001	<0.001
B vs O	<0.001	<0.001	<0.001
C vs N	0.001	0.002	<0.001
C vs M	<0.001	<0.001	<0.001
C vs O	<0.001	<0.001	<0.001
N vs M	0.327	0.179	0.610
N vs O	0.354	0.535	0.981
M vs O	0.420	0.425	0.470

**Figure 3:**
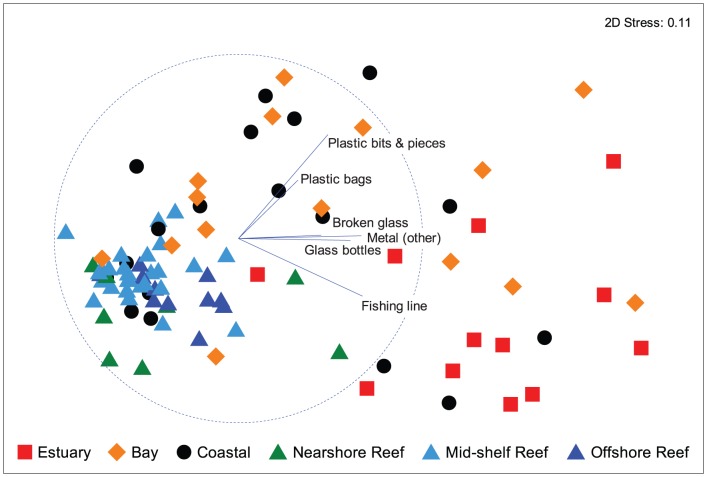
Non-metric multidimensional scaling ordination of debris items recorded from 120 sites. Data points represent averages of transects deployed at each site. Vectors are displayed for debris items that were consistently ranked highly in SIMPER analyses of differences among habitats. The vector line indicates the plane of increasing abundance, and the length relative to the circle indicates the strength of the correlation (Spearman).

The nMDS plot of patterns of debris structure (Fig. 3) did not show clear clustering of sites based on habitat: nevertheless, there were some obvious trends. With the exception of the 2 sites at Cook Island (green triangles to the lower right of the main cluster within the circle), all nearshore, mid-shelf and offshore sites group to the left of the plot and are typified by low abundances of all of the discriminant debris categories (Fig. 3). PERMANOVA indicated highly significant effects for both habitat type (P<0.001) and site nested in habitat type (P<0.001). Subsequent pairwise tests (Table 1) revealed that all habitat types were significantly different from each other with the exception of bay vs coast, and all comparisons amongst reefs. Most estuarine sites appear in the lower right of the plot and are typified by high abundance of fishing line, metal objects, glass bottles and glass fragments. While there is considerable variation among sites within bays, those in the upper plot are typified by high abundance of plastic bags and plastic fragments. Sites within the coastal category are highly variable, being spread amongst all other habitat types, but many of these were also discriminated by comparatively high abundances of plastic bags and other plastic items (Fig. 3).

## Discussion

This study provides the first overview of differences in the abundance and type of debris in 6 prominent coastal and marine habitat types along 1000 km of the coast of eastern Australia. Estuarine and coastal systems are widely recognised as being amongst the most heavily threatened natural ecosystems worldwide [40], [41]. This study confirms the significantly higher prevalence of anthropogenic debris in estuaries and bays compared to other coastal and marine habitats in NSW. These findings provide unambiguous guidelines for prioritisation of mitigation, monitoring and management efforts at a statewide scale. The fact that the most affected habitats have high natural, recreational and economic values [40] suggests that there is some urgency in developing and implementing strategies to reduce potential impacts. While this may be difficult to implement over the large spatial scales of the study, location-specific strategies, in response to some obvious threats revealed in this study (see below), should be more readily effected. A critical step in targeted management is the identification of primary sources of debris. In this case, and reflecting the findings of many other studies worldwide, fishing-related debris was the most prevalent in all subtidal habitats (38% of total items), with fishing line being the most abundant item (27% of total items). However, it is highly likely that the number of fishing-related items is under-represented in the data, as only items directly related to fishing activities were allocated to that category. For example, bricks found at reef sites (a total of 40 across all sites) are most likely to be remnants of fishing traps (bricks are used to weight the corners of traps - pers. obs.). In addition, at some popular fishing sites, it is highly likely that other items (such as clothing, food and drink items) result from fishing-related activities [42], [43].

Putting the results into a global perspective, while debris loads across the study were highly variable at the scale of sites (mean range from 0–77.5 transect^−1^) and transects (0–218 items transect^−1^), densities at the most-affected sites were amongst the highest recorded from shallow-water habitats worldwide (equivalent to 620 items 1000 m^−2^ in the Nambucca River Estuary and 528 items 1000 m^−2^ at the Pipeline) [reviewed in [9]). Values exceeding this have only previously been published for heavily populated areas such as Indonesia [44] and the West Indies [45]. However, this needs to be balanced by two specific observations: ∼45% of the transects deployed during the study contained no debris (all of these transects occurred >150 m from shore with most being in the mid-shelf and offshore category); with the exception of the 2 bays that were evaluated, only a small proportion of debris items were of domestic (household) origin. So, despite some isolated, very high loads, our results suggest that there is generally good waste management in NSW and that much of the debris found in coastal habitats is deposited *in situ* (e.g. through boating and fishing activities).

Although not analysed specifically in this study, it is clear that ease of access, and consequent *in situ* deposition, strongly contributed to comparative debris loads. The items primarily differentiating estuaries and bays from other habitats were fishing line (almost always entangled around features of the benthos or benthic taxa), metal objects, glass bottles, and glass fragments. While the 2 latter items may be moved by strong tidal currents or scour following extreme weather events, these discriminatory items are generally less mobile than many of the other debris items recorded during the study, and are likely to have been deposited close to where they were found. Indeed, the sites that had the highest debris loads (Nambucca River Estuary and the Pipeline, Port Stephens) were at popular fishing and recreation locations, with access facilitated by a boardwalk and a breakwater, respectively. The sites with the highest debris load in the nearshore, mid-shelf and offshore categories were associated with moorings (Cook Island North - mean  = 6.5 transect^−1^; ex-HMAS Adelaide - mean  = 6.0 transect^−1^; Cook Island South - mean  = 4.8 transect^−1^) which provide a clear focus for boating and recreational activities that generate marine debris [42], [43].

That the highest debris loads occurred in regional areas of NSW rather than adjacent to major population centres, such as Sydney, requires some interpretation. While surveys were conducted in the Sydney region, they were mainly carried out from boats and targeted reefs within Sydney Harbour rather than accessible shore sites. Although 3 shore sites were surveyed, these were not in areas with the highest usage rates. Indeed, access to the latter is often very difficult due to the risk presented by boat traffic and conflict with fishers. It is, therefore, highly likely that there are many sites with much greater debris loads than recorded here, and that these need to be assessed in any future programs.

By far the majority of studies of benthic debris have been one-off in nature and very few have assessed accumulation rates (but see [15], [46]). However, accumulation studies are a natural and important extension of assessments of standing stock [22] and provide important information to fine-tune management strategies through, for example, targeted clean up [46]. As has been well documented for intertidal habitats [47], [48] it is highly likely that accumulation rates are non-linear and dependent on a range of factors. For example, seasonal effects of storms that flush catchments, and influx of visitors engaged in boating and fishing activities, are likely to strongly impact accumulation rates. This initial spatial overview of debris loads provides an important baseline against which to measure accumulation rates, and longer-term monitoring to quantify this has already commenced at the heavily contaminated identified in this program (e.g. the Nambucca River Estuary; The Pipeline, Port Stephens; Cook Island).

From the perspective of management at a local scale, observations from a number of sites are worth mentioning in greater detail. Firstly, each of the 3 most contaminated reef sites are putatively protected from fishing activities by fishing closures (2 sites at Cook Island, a gazetted Aquatic Reserve in northern NSW; the artificial reef, ex-HMAS Adelaide on the central NSW coast which is also a designated Reserve with a fishing closure). Collectively, transects at these sites contained 58 debris items, of which 40 were directly related to fishing (mostly fishing line). Clearly, the presence of these items identifies lack of compliance as an issue contributing directly to debris loads. An important consideration in inferring non-compliance is that items did not pre-date protection. In the case of the ex-HMAS Adelaide, protection occurred at the time of its sinking. Protective legislation for Cook Island was gazetted in 1998 and while 2 pieces of fishing line were observed with a substantial coating of encrusting coralline algae, we are confident that by far the majority of items were deposited since 1998.

Unfortunately, non-compliance with fishing closures appears to be an ubiquitous issue both within this study and elsewhere: most of the sites surveyed within sanctuary zones in the Solitary Islands Marine Park contained fishing-related debris; fishing-related debris in the Florida Keys has been found to be as prevalent in areas closed to fishing as in unrestricted areas [24]. At Cook Island, the majority of fishing line was found entangling colonies of scleractinian coral, which is a dominant feature of benthic communities at many sites in northern NSW [27], [49], [50]. Given the demonstrated association between monofilament entanglement and the morbidity and mortality of coral and other sessile invertebrates [23], [24], [27], [51], this observation warrants further investigation by the managing authority.

**Figure 4:**
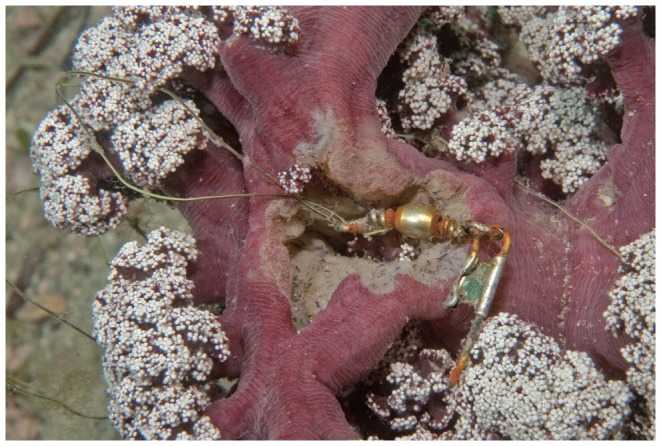
Direct impact of fishing activities on a colony of the geographically restricted soft coral *Dendronephthya australis* at the Pipeline, Port Stephens.

One other site to single out is the Pipeline, Port Stephens. This site is within the Port Stephens Great-Lakes Marine Park (PSGLMP), but in a zone where fishing is permitted. The close proximity to a car park and breakwater make it a very popular site for fishing: consequently, fishing-related items comprised 228 of the 329 items recorded from the 2 sites surveyed at this location. This site was recently identified as having very high conservation value [52] as it contains one of only a few populations of a geographically restricted, habitat-forming soft coral (*Dendronephthya australis*). These soft corals provide habitat for a high diversity of biota including juveniles of commercially important fish and a wide range of invertebrate species, including species at the southern limits of their distribution range [52], [53]. Consequently, this habitat is important for regional biodiversity conservation [54], and may become more so in the near future as a refuge for taxa driven poleward by a changing climate [55]. Observations made during surveys at this site unambiguously demonstrate direct impacts of fishing line (Fig. 4) and strongly support the need for immediate improvement in protective measures.

This broad assessment of marine debris loads across NSW has provided data that will help to set priorities for resourcing clean-up and other mitigation strategies, by habitat type, across the state, whilst identifying pressing management issues that need to be addressed at local scales. The data collected to date also provide an important baseline against which accumulation rates, and the success of mitigation measures, can be objectively assessed. Importantly, the study also highlights the very valuable role of citizen scientists in the provision of data to promote sustainable management of coastal habitats and resources: consequently, we strongly advocate the continued engagement of researchers and managers with suitably motivated volunteers. Debris surveys represent an ideal choice of topic given, for example, the restricted scope for issues such as mistaken identity. However, provided that adequate training is given, appropriate quality assurance/quality control procedures are rigorously enforced, and broader programs are designed appropriately, there is ample evidence that volunteers can provide highly relevant data that facilitate high-end scientific outcomes [56].
